# Reimagining tuberculosis elimination in India: diagnostics, drug resistance, and digital health strategies

**DOI:** 10.3389/fepid.2026.1868261

**Published:** 2026-06-29

**Authors:** Sneha Jha, Loka Bikash Chaliha, Kshipra Pandey, Ritu Patel

**Affiliations:** Department of Bioscience, Indrashil University, Rajpur, Gujarat, India

**Keywords:** anti-tuberculosis medications, drug-resistance, eradication of TB, health policies, *Mycobacterium tuberculosis*, tuberculosis

## Abstract

Tuberculosis (TB) remains a major global public health challenge and one of the leading infectious causes of mortality worldwide, with India contributing approximately 26% of the global TB burden. Despite substantial progress under the National Tuberculosis Elimination Programme (NTEP), India's efforts to achieve the ambitious 2025 TB elimination target continue to face major challenges due to the COVID-19 pandemic, multidrug-resistant tuberculosis (MDR-TB), healthcare disparities, and limitations in surveillance and reporting systems. This review provides a comprehensive overview of the epidemiology of TB in India and critically evaluates the impact of COVID-19, drug resistance, healthcare accessibility, and emerging diagnostic technologies on TB control efforts. A structured literature search was conducted using PubMed, Scopus, and Google Scholar, along with reports from the World Health Organization and the Government of India. The reviewed evidence indicates that the COVID-19 pandemic significantly disrupted TB diagnosis, treatment, surveillance, and healthcare accessibility, leading to declines in case notification and the possible accumulation of undiagnosed TB cases. In addition, the increasing burden of MDR/RR-TB, fragmented private healthcare systems, underreporting, and unequal access to diagnostics and treatment continue to hinder progress toward TB elimination. Although advancements including molecular diagnostics, digital surveillance platforms such as Nikshay, AI-assisted screening, and patient-support programmes have strengthened TB control strategies, important challenges related to implementation, infrastructure, scalability, and antimicrobial stewardship remain unresolved. This review highlights the need for integrated and patient-centred TB control strategies that combine rapid diagnostics, strengthened surveillance, public–private healthcare integration, health systems strengthening, and interventions addressing broader socioeconomic determinants such as poverty, malnutrition, and healthcare inequity. Coordinated multisectoral approaches will be essential for accelerating TB elimination efforts in India in the post-COVID era.

## Graphical Abstract

**Figure:**
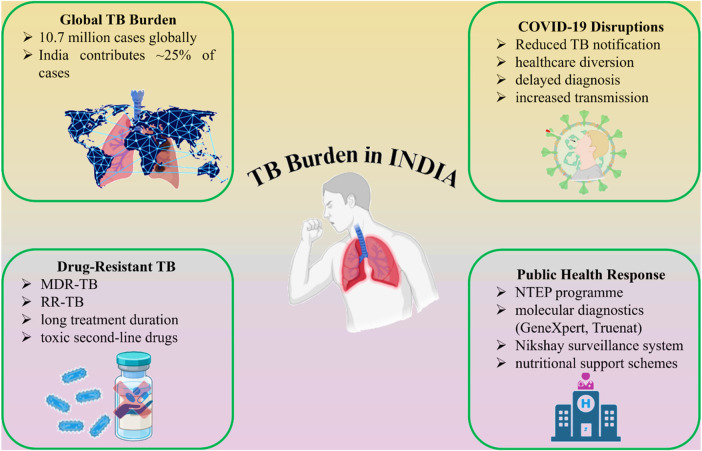
Conceptual overview of factors influencing tuberculosis elimination in India. The figure illustrates the global burden of tuberculosis, the disruptive impact of the COVID-19 pandemic on TB control programmes, the growing challenge of drug-resistant tuberculosis, and the role of national public health interventions such as the National Tuberculosis Elimination Programme (NTEP) in addressing these challenges.

## 1 Introduction

Tuberculosis (TB) remains one of the leading infectious causes of morbidity and mortality worldwide despite the availability of effective diagnostic and therapeutic interventions. TB is caused by *Mycobacterium tuberculosis* and primarily affects the lungs, although extrapulmonary manifestations are also common. The disease spreads through airborne transmission when infected individuals expel bacteria through coughing, sneezing, or speaking. According to the World Health Organisation (WHO), approximately 10.7 million people developed TB globally in 2024, including men, women, and children, making it one of the leading causes of mortality from a single infectious pathogen worldwide. Despite being a preventable and curable disease, TB caused approximately 1.2 million deaths globally, emphasising the persistent burden of this infection in many parts of the world (1).

Globally, TB remains concentrated within a limited number of high-burden countries. More than two-thirds of global TB cases are reported from eight countries, including India, Indonesia, China, the Philippines, Pakistan, Nigeria, Bangladesh, and the Democratic Republic of Congo, with India contributing the largest share of the global burden (approximately 25%–26%) (2). These regions continue to face multiple challenges, including delayed diagnosis, poverty, malnutrition, overcrowding, and limited access to healthcare, all of which contribute to sustained disease transmission and poor treatment outcomes.

India remains central to global TB elimination efforts and continues to report the highest number of TB cases worldwide. Recent reports indicate that approximately 2.7 million TB cases were reported in India in 2023, accounting for nearly one-quarter of global TB cases despite considerable progress in reducing incidence over the past decade. The incidence rate of TB in India declined from 237 cases per 100,000 population in 2015 to approximately 187 cases per 100,000 population in 2024, representing a reduction of about 21% (3, 4). However, major challenges including population density, socioeconomic disparities, healthcare inequalities, and uneven access to diagnostics and treatment continue to hinder TB elimination across the country. In addition to biomedical barriers, gaps in health systems governance, fragmented healthcare delivery, and limitations in surveillance and reporting systems continue to influence TB transmission, diagnosis, and treatment outcomes in India.

To address the TB epidemic, the Government of India implemented the National Tuberculosis Elimination Programme (NTEP) with the ambitious goal of eliminating TB by 2025, five years ahead of the global target established under the WHO End TB Strategy and the Sustainable Development Goals (SDGs). The programme focuses on early diagnosis, universal drug-susceptibility testing, improved treatment regimens, and strengthened surveillance systems such as the Nikshay digital platform, which enables real-time monitoring of TB cases and treatment outcomes across the country (5, 6). These initiatives have substantially improved treatment coverage, molecular diagnostic accessibility, and TB case notification over the past decade. However, the emergence of the coronavirus disease 2019 (COVID-19) pandemic significantly disrupted TB control programmes worldwide. The pandemic diverted healthcare resources, interrupted diagnostic and treatment services, reduced healthcare accessibility, and led to substantial declines in TB case notifications in many countries (7). Lockdowns, healthcare system overload, and similarities in respiratory symptoms between TB and COVID-19 further complicated timely diagnosis and treatment. Consequently, many undiagnosed or untreated TB cases may have contributed to increased community transmission during the pandemic period.

Another major challenge threatening TB elimination efforts is the growing burden of drug-resistant tuberculosis (DR-TB), particularly multidrug-resistant TB (MDR-TB) and rifampicin-resistant TB (RR-TB). India accounts for a substantial proportion of global drug-resistant TB cases, while prolonged treatment duration, high treatment costs, treatment-related toxicity, and inconsistent treatment adherence continue to complicate disease management (8). Furthermore, underreporting from the private healthcare sector and non-standardised treatment practices remain important barriers to effective DR-TB surveillance and control.

Although substantial progress has been achieved through improved diagnostics, expanded treatment access, and strengthened public health interventions, achieving TB elimination in India remains highly challenging. Multiple interconnected factors, including drug resistance, healthcare disparities, socioeconomic determinants, delayed diagnosis, and disruptions caused by global health emergencies, continue to hinder progress. Therefore, this review aims to summarise the current epidemiological status of tuberculosis in India and critically evaluate the impact of the COVID-19 pandemic and other contributing factors affecting TB control and elimination efforts. In addition, the review highlights emerging challenges such as drug-resistant TB, gaps in healthcare systems, and the need for integrated, technology-driven, and patient-centred public health strategies to accelerate progress toward TB elimination.

## 2 Literature search strategy and study selection

A structured literature search was conducted to identify relevant studies related to tuberculosis epidemiology, diagnostics, drug resistance, treatment strategies, and tuberculosis elimination efforts in India. Electronic databases including PubMed, Scopus, and Google Scholar were searched for articles published between January 2015 and March 2025. Additional data and policy documents were retrieved from the World Health Organization (WHO), the Ministry of Health and Family Welfare, Government of India, and reports from the NTEP.

The search strategy included combinations of keywords such as “tuberculosis”, “India”, “drug-resistant tuberculosis”, “MDR-TB”, “COVID-19 and tuberculosis”, “tuberculosis diagnostics”, “GeneXpert”, “Truenat”, “NTEP”, “Nikshay”, and “tuberculosis elimination”.

Studies were included if they: (i) focused on tuberculosis epidemiology, diagnosis, treatment, public health strategies, or drug resistance; (ii) discussed tuberculosis burden or elimination efforts in India; (iii) addressed the impact of COVID-19 on tuberculosis control; and (iv) were published in English.

Studies were excluded if they were duplicate records, conference abstracts without full text, non-English publications, or articles not directly relevant to the objectives of this review. Relevant studies were screened based on titles, abstracts, and full-text assessment. The selected literature was narratively synthesised to provide an updated overview of tuberculosis elimination challenges, diagnostic advances, drug resistance, and public health interventions in India.

## 3 Global and national burden of Tuberculosis

### 3.1 Global epidemiology of tuberculosis

**Figure 1:**
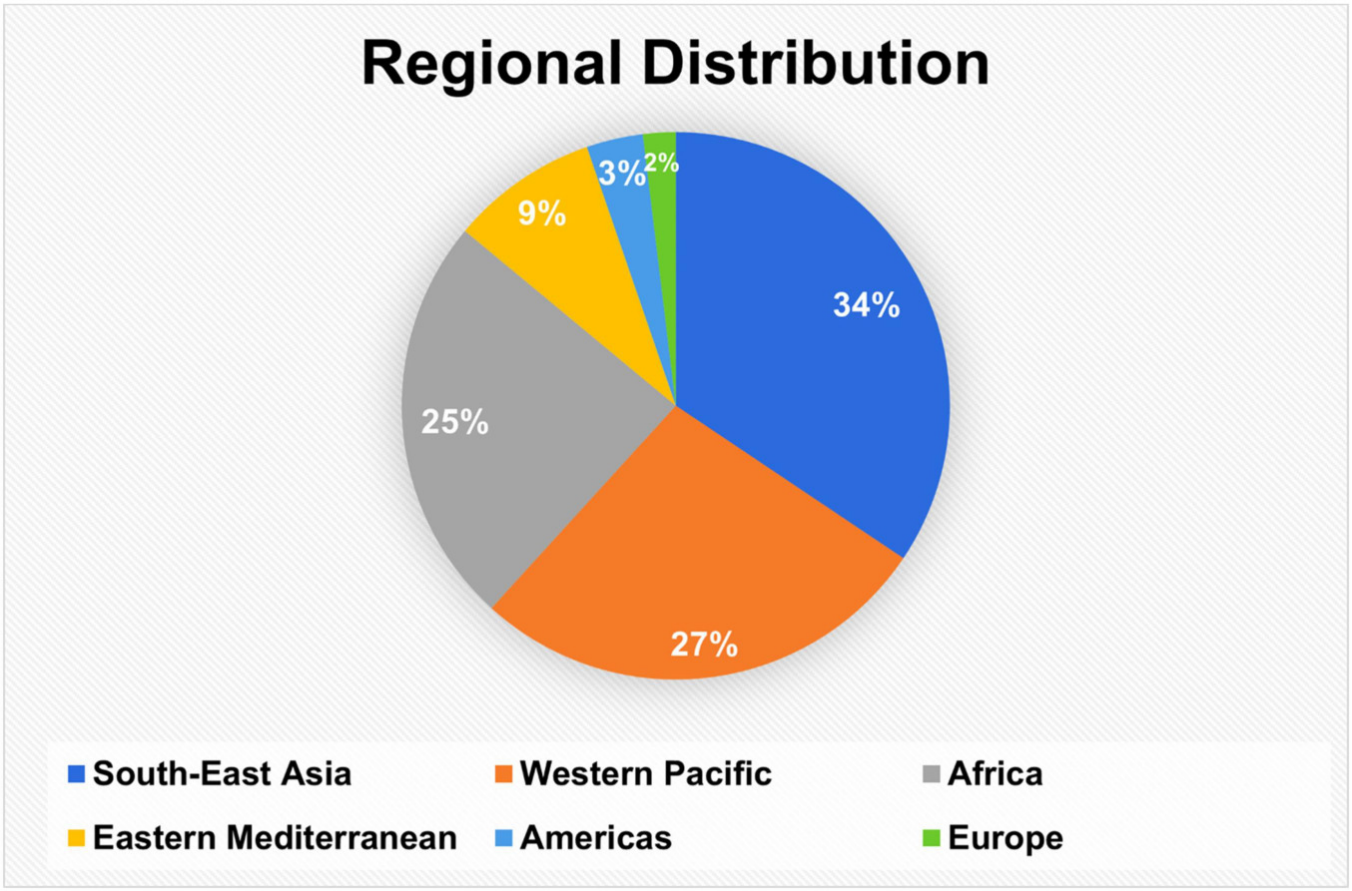
Regional distribution of the global tuberculosis burden based on data reported in the WHO global Tuberculosis report 2024. Data adapted from the World Health Organization (WHO) (1, 6, 9).

It is estimated that approximately 10.7 million people developed tuberculosis (TB) worldwide in 2024, corresponding to an incidence of 131 cases per 100,000 population (1, 2). Among these cases, nearly 5.8% occurred in individuals living with HIV infection (1). The global distribution of TB remains highly uneven, with the WHO South-East Asia region contributing the largest proportion of cases (34%), followed by the Western Pacific (27%) and Africa (25%), while lower proportions were reported from the Eastern Mediterranean (8.6%), the Americas (3.3%), and Europe (1.9%) (1). As illustrated in Figure 1, the concentration of TB burden within a limited number of high-burden regions and countries highlights major global inequities in healthcare access, socioeconomic conditions, and public health infrastructure.

Notably, five Asian countries alone contribute more than half of the global TB burden, emphasising the need for region-specific control strategies and targeted resource allocation. The persistence of high TB incidence in these settings is strongly associated with poverty, malnutrition, overcrowding, limited access to rapid diagnostics, and weak healthcare systems, all of which continue to sustain transmission dynamics (1, 9). Although the estimated number of TB cases declined for the first time since the COVID-19 pandemic after consecutive increases between 2020 and 2023, TB still caused approximately 1.2 million deaths globally in 2024, including around 167,000 deaths among people living with HIV infection (1). These findings reinforce that TB remains not only a biomedical challenge but also a major socioeconomic and health systems issue requiring coordinated global and national policy responses. Consequently, TB has re-emerged as the leading infectious cause of mortality worldwide, surpassing COVID-19 in recent global estimates (Figure 1) (4, 9).

## 4 Tuberculosis burden in India

India continues to report the highest tuberculosis (TB) burden globally, accounting for approximately 26% of all global TB cases in 2024. Regional distribution of the global tuberculosis burden based on data reported in the WHO Global Tuberculosis Report 2024. Data adapted from the World Health Organization (WHO) (1). India has achieved a greater reduction in TB incidence and mortality compared with the global average, with incidence declining from 237 cases per 100,000 population in 2015 to approximately 187 cases per 100,000 population in 2024, representing a reduction of about 21% (3, 10). This rate of decline exceeds the global average reduction of nearly 12%, highlighting the impact of intensified TB control strategies implemented under the NTEP.

Despite this progress, substantial regional disparities continue to influence TB transmission and treatment outcomes across India. States such as Uttar Pradesh, Maharashtra, Bihar, Madhya Pradesh, and Rajasthan contribute a major proportion of the national TB burden due to high population density, urban overcrowding, poverty, and healthcare inequalities. In contrast, several northeastern and tribal regions, including Nagaland and Mizoram, report comparatively higher per capita TB prevalence, which may reflect limited healthcare accessibility, delayed diagnosis, malnutrition, and weaker healthcare infrastructure (11). These regional differences highlight broader socioeconomic and health systems challenges that continue to hinder equitable TB control and surveillance across the country.

**Figure 2:**
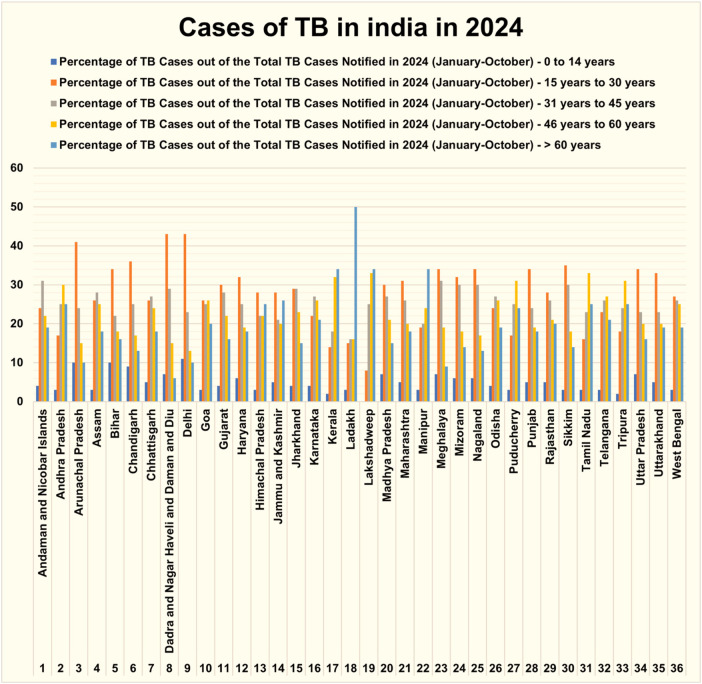
State-wise distribution of notified tuberculosis cases under the national Tuberculosis elimination programme (NTEP) in India during 2024. Data adapted from Nikshay Dashboard and Government of India reports (1, 6).

As illustrated in Figure 2, the age-wise distribution of TB cases notified across Indian states and union territories in 2024 demonstrates that the highest proportion of TB cases occurs among economically productive age groups, particularly individuals between 15 and 45 years of age. This trend suggests substantial socioeconomic implications, including loss of productivity, increased healthcare expenditure, and long-term financial burden on affected households. In addition, variations in age-specific TB distribution across different states may reflect differences in population demographics, healthcare accessibility, nutritional status, urbanisation, and public health surveillance systems. The figure further emphasises the need for region-specific and population-targeted TB control interventions rather than uniform national approaches.

The COVID-19 pandemic significantly disrupted TB control programmes during 2020 and 2021, resulting in an estimated 25%–30% decline in TB case notifications compared with pre-pandemic levels (12). Diversion of healthcare resources, interruptions in diagnostic services, reduced healthcare accessibility, and overlapping respiratory symptoms between TB and COVID-19 collectively contributed to delayed diagnosis and underreporting of TB cases. These disruptions may have led to the accumulation of undiagnosed TB cases and continued community-level transmission during the pandemic period.

Post-pandemic data indicate substantial recovery in TB detection and treatment services following expansion of rapid molecular diagnostics such as GeneXpert and Truenat, implementation of the Nikshay digital surveillance platform, and active case-finding campaigns targeting vulnerable populations (13). By 2024, approximately 92% of TB patients in India underwent rifampicin-resistance testing compared with the global average of 83%, while treatment coverage and treatment success rates also improved substantially (14). However, despite these achievements, persistent challenges including underreporting from the private healthcare sector, uneven healthcare infrastructure, and delayed diagnosis in rural and underserved settings continue to limit progress toward sustainable TB elimination in India.

## 5 Impact of COVID-19 on tuberculosis control

COVID-19 has posed unique clinical challenges where patients with pulmonary TB were more vulnerable to severe COVID-19 outcomes due to impaired lung function, whereas COVID-19 infection progressed TB advancement by a weakened immune system. Indian studies have shown higher mortality rates and prolonged hospital stays among coinfected patients compared to those with either of the diseases alone (12). Clinicians have also struggled with problems in differentiating between TB and COVID-19 symptoms, as both diseases present with cough, fever and respiratory distress, which has resulted in diagnostic delays and mismanagement.

### 5.1 Decline in TB case notifications during the pandemic

**Figure 3:**
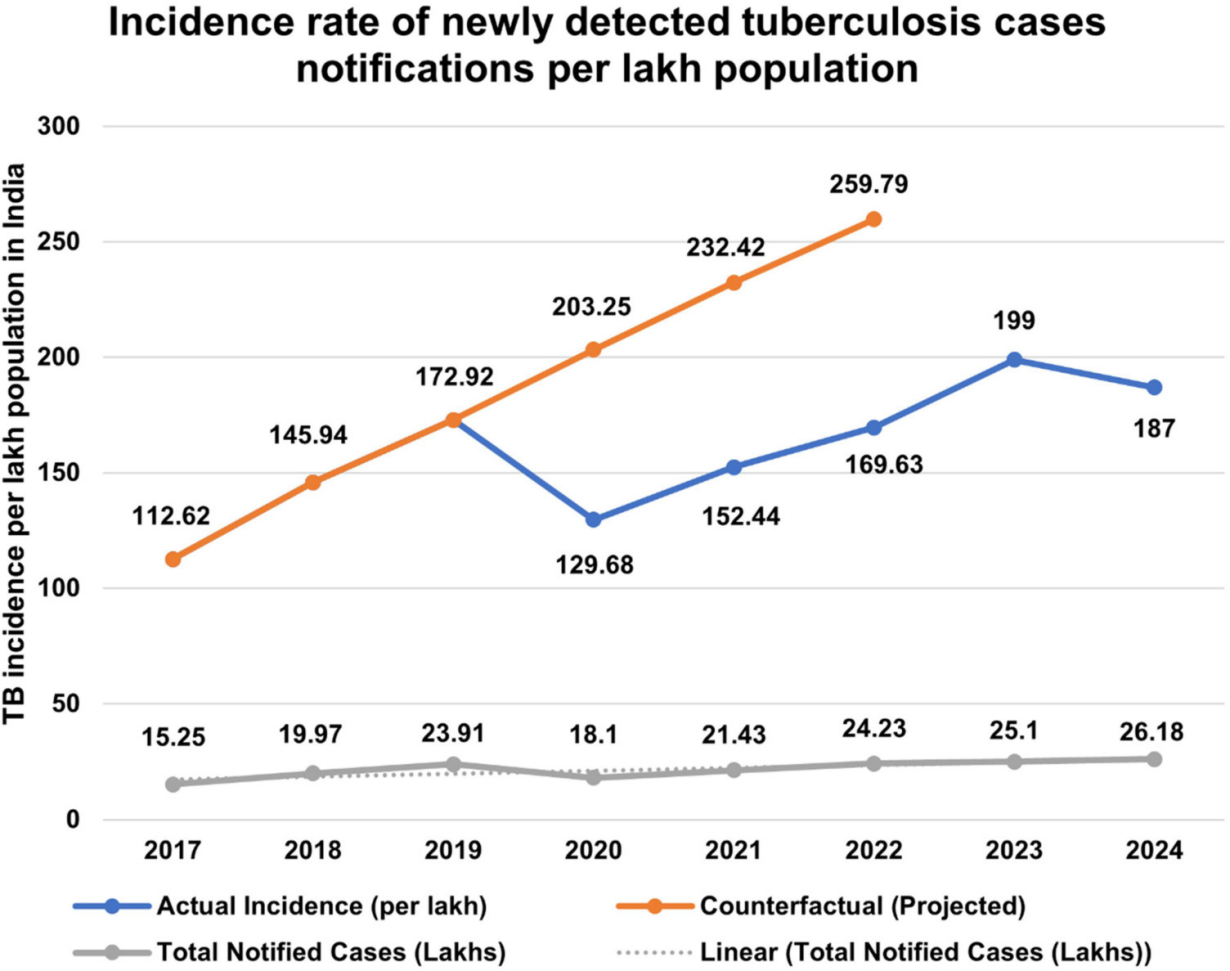
Temporal trends in tuberculosis incidence and notification in India (2017–2024), illustrating the divergence between observed and counterfactual projections. A substantial drop in reported incidence during 2020 reflects COVID-19–related disruptions in TB detection and reporting systems, while the post-pandemic recovery indicates restoration of surveillance and diagnostic capacity. The gap between projected and actual trends suggests a hidden burden of undiagnosed TB during the pandemic period (4, 5).

India has shown a significant reduction, falling by nearly 18% in 2020, and alone reported a 25%–30% decline compared to pre-pandemic levels. This decline was because of various factors, including reduced patient visits to healthcare facilities, misdiagnosis due to overlapping respiratory symptoms with COVID-19, and the diversion of healthcare staff and resources to pandemic management (6). The unrecognised TB cases during this period likely contributed to a significant increase in the transmission, as untreated individuals remained infectious. The divergence between the observed and counterfactual projections shown in Figure 3 further suggests the presence of a substantial hidden burden of undiagnosed TB cases during the COVID-19 period. This gap highlights how disruptions in healthcare access, reduced diagnostic capacity, and diversion of public health resources may have masked the true epidemiological burden of TB rather than reflecting an actual reduction in disease transmission. The delayed identification of these missed cases may continue to influence transmission dynamics and drug resistance patterns in the post-pandemic period. Notifications recovered in 2022–2024 were supported by the expansion of digital surveillance systems such as Nikshay, which enabled real-time monitoring of TB cases, and by the integration of TB screening in COVID-19 testing centres (6).

### 5.2 TB–COVID coinfection and clinical implications

The COVID-19 pandemic severely disrupted TB diagnostic and treatment services in India due to coinfection. During the peak of the pandemic, healthcare infrastructure was reallocated to COVID-19 treatment, leading to reduced availability of TB-specific resources such as diagnostic laboratories, hospital beds, and trained personnel (Figure 3) (7). Molecular diagnostic platforms like GeneXpert and Truenat, which are necessary for rapid TB detection, were diverted for COVID-19 diagnosis, resulting in delays in TB detection. Lockdowns and restrictions on movement further limited patient access to healthcare facilities, causing inadequate treatments, which have increased the risk of MDR-TB (8).

## 6 Drug-resistant tuberculosis

(DR-TB) represents one of the most serious threats to global tuberculosis control and remains a major barrier to India's TB elimination programme (Table 1). The rise of multidrug-resistant TB (MDR-TB) and rifampicin-resistant TB (RR-TB) is challenging treatment strategies, increasing healthcare costs, and reducing treatment success rates. Despite advancements in diagnostics and new drug regimes, despite advances in diagnostics and newer treatment regimens, India continues to report one of the highest burdens of DR-TB globally.

**Table 1:** WHO classification of drug-resistant tuberculosis types [world health organisation, 2025 (1–3)].

Types of drug-resistant TB	Definition
Mono-resistant TB	TB caused by strains resistant to one first-line anti-tuberculosis drug only.
Poly-resistant TB	TB caused by strains resistant to more than one first-line anti-tuberculosis drug, other than the combination of isoniazid and rifampicin.
Multidrug-resistant TB (MDR-TB)	TB caused by strains resistant to at least isoniazid and rifampicin, the two most effective first-line anti-tuberculosis drugs.
Pre-extensively drug-resistant TB (pre-XDR-TB)	MDR/RR-TB with additional resistance to any fluoroquinolone.
Extensively drug-resistant TB (XDR-TB)	MDR/RR-TB with additional resistance to any fluoroquinolone and at least one additional Group A drug (bedaquiline or linezolid).

### 6.1 MDR-TB and RR-TB epidemiology

Multidrug-resistant TB (MDR-TB) is defined as resistance to at least isoniazid and rifampicin, while RR-TB refers to resistance to rifampicin. In 2023, approximately 63,000 MDR/RR-TB cases were reported in India, representing a substantial proportion of the global drug-resistant TB burden (1, 6). The epidemiology of DR-TB in India is highly influenced by regional disparities, with urban centres such as Mumbai and Delhi reporting high numbers of MDR-TB cases due to high population rates and better diagnostics. In contrast, rural and tribal regions often have underreported cases due to limited access to drug susceptibility testing. The proportion of primary MDR-TB cases where resistance is acquired through direct transmission rather than treatment failure has been rising significantly, which suggests that inter-community spreading of different strains (15).

### 6.2 Causes of drug resistance

Drug resistance in *Mycobacterium tuberculosis* can be classified into primary resistance, which occurs when individuals are infected directly with resistant strains of TB (16), whereas secondary resistance occurs when patients with drug-sensitive TB acquire resistance during treatment, which can usually be due to incomplete therapy, poor adherence, or insufficiently implemented (17).

**Figure 4:**
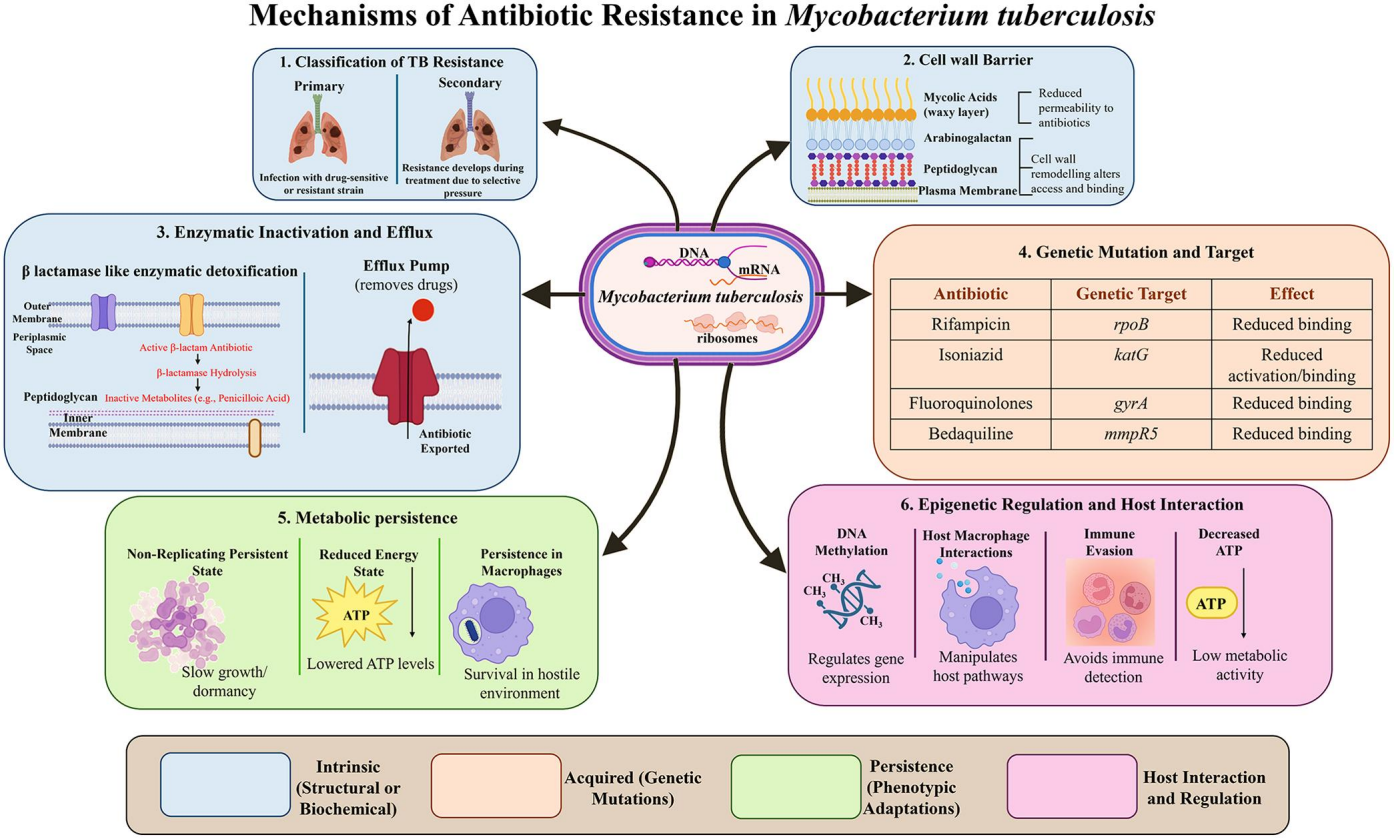
Mechanisms of antibiotic resistance in *Mycobacterium tuberculosis*. The schematic illustrates key resistance pathways, including target site mutations affecting drugs such as rifampicin (rpoB), isoniazid (katG/inhA), and fluoroquinolones (gyrA/gyrB), as well as intrinsic resistance mechanisms such as reduced cell envelope permeability and active drug efflux pumps. Additionally, inhibition of critical bacterial processes such as RNA synthesis and protein synthesis is shown, highlighting how genetic mutations and physiological barriers collectively contribute to reduced drug efficacy.

The mechanistic innate features of drug resistance include three factors: an impermeable cell wall, slow-metabolism mechanisms, and numerous efflux pumps. *Mycobacterium tuberculosis* exhibits drug resistance primarily due to its unique cell wall structure, which comprises mycolic acids, Wax-D, cord factors, and a hydrophilic arabinogalactan layer covalently linked to peptidoglycan. These layers create a formidable lipid barrier that inhibits the entry of both hydrophobic and hydrophilic antibiotics, resulting in slow drug accumulation followed by enzymatic detoxification via β-lactamases and other cellular mechanisms. Moreover, the outer membrane channel protein CpnT facilitates selective nutrient uptake while conferring susceptibility patterns, whereas CpnT mutants demonstrate enhanced resistance to multiple antitubercular agents, including nitric oxide (18). Slow metabolic processes are generally exacerbated by stress-induced triacylglycerol accumulation under hypoxia, acidic pH, or iron limitation, resulting in depletion of acetyl-CoA, which is essential for the TCA cycle, thereby rendering antibiotics unstable (e.g., carbapenems) and rendering them ineffective before bacterial replication (19). Additionally, numerous multidrug efflux pumps spanning the inner/outer membranes actively export antibiotics and are regulated by specific protein systems, serving dual roles in physiology (nutrient/toxin transport) and resistance (Figure 4) (20).

At the molecular level, resistance arises from genetic mutations in specific bacterial genes that alter drug targets or metabolic pathways, reducing the efficacy of anti-TB drugs. For example, rpoB mutations that confer rifampicin resistance; katG and inhA mutations cause isoniazid resistance; pncA mutations lead to pyrazinamide resistance; embB mutations are linked to ethambutol resistance; and gyrA and gyrB mutations result in fluoroquinolone resistance (15, 21, 22).

## 7 Challenges in treatment and management

Treating DR-TB is significantly more difficult than managing drug-sensitive TB due to multiple clinical, economic, and social challenges. The treatment duration for MDR-TB is prolonged, often requiring 18–24 months of therapy compared to just 6 months for drug-sensitive TB, which increases the risk of treatment fatigue and abandonment (23). The toxicity and side effects of second-line drugs such as kanamycin, cycloserine, and ethionamide are severe, ranging from hearing loss and psychiatric disturbances to gastrointestinal complications, all of which discourage adherence (24, 25). In addition, the cost of treatment is nearly ten times higher than that of drug-sensitive TB, placing a heavy burden on patients and straining public health budgets (26, 27).

Although newer drugs such as bedaquiline, delamanid, and pretomanid have improved treatment outcomes, access remains uneven across India, particularly in rural and tribal regions (28). Even with these advances, treatment success rates remain low, averaging around 60% for MDR-TB compared with approximately 90% for drug-sensitive TB (1). Infrastructure gaps further complicate management, as specialised MDR-TB centres remain concentrated in urban settings, limiting access for underserved populations. Beyond clinical barriers, stigma, financial hardship, discrimination, and employment insecurity continue to reduce treatment adherence and worsen patient outcomes (29).

**Figure 5:**
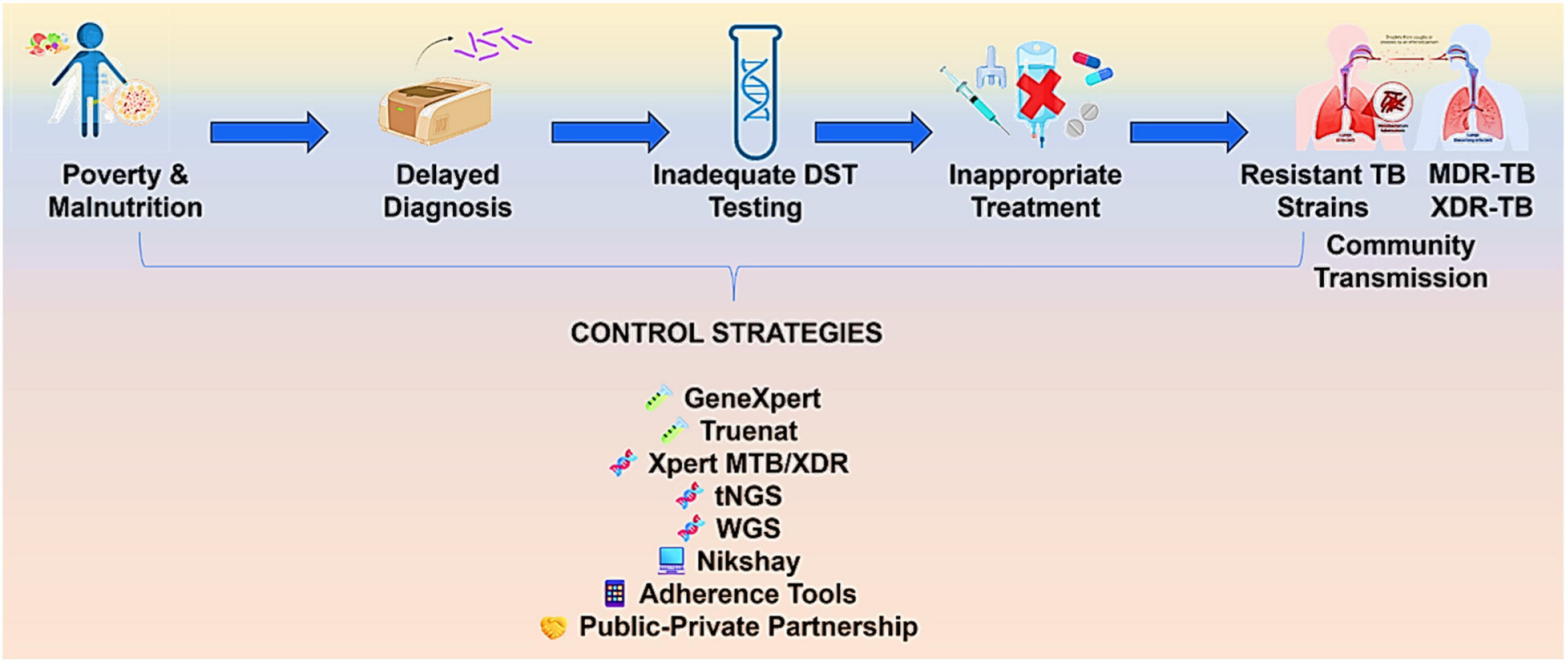
Pathways contributing to the emergence and transmission of drug-resistant tuberculosis (DR-TB). Delayed diagnosis, inadequate drug susceptibility testing, inappropriate treatment, and poor treatment adherence facilitate the development of resistant *Mycobacterium tuberculosis* strains, leading to MDR-TB and XDR-TB. Advanced diagnostics and digital health interventions can help interrupt this pathway and improve tuberculosis control.

As illustrated in Figure 5, the emergence and spread of antimicrobial resistance are not driven solely by inappropriate TB treatment practices but are also influenced by broader environmental, agricultural, and public health factors. Excessive antibiotic use in humans and livestock, environmental contamination, and transmission through food systems collectively contribute to resistance development and dissemination. This highlights the importance of adopting a broader One Health perspective when addressing drug-resistant TB, recognising the interconnected roles of human health, animal health, and environmental exposure in antimicrobial resistance dynamics. Inadequate treatment regimens, poor adherence, and weak antimicrobial stewardship may further accelerate the progression from MDR-TB to extensively drug-resistant TB (XDR-TB), making disease management substantially more difficult and limiting future therapeutic options.

## 8 Diagnostic advances in tuberculosis detection

### 8.1 Conventional diagnostic methods

Conventional diagnostic methods are crucial for detecting tuberculosis (TB), particularly in resource-limited settings. These methods include phenotypic drug susceptibility testing (DST), smear microscopy, acid-fast staining, fluorescence microscopy, tuberculin skin testing, and chest x-rays (Table 2) (28). While they are relatively inexpensive and widely accessible, their primary limitations include sensitivity, specificity, and turnaround time. Due to these limitations, there has been significant development in molecular and diagnostic technologies (30).

**Table 2:** Comparison of major molecular diagnostic assays for tuberculosis detection, including geneXpert, truenat, and conventional PCR. The table summarises their underlying technologies, turnaround time, target genes, sensitivity in smear-negative cases, key strengths, limitations, and scale-up status under India's National Tuberculosis Elimination Programme (NTEP).

Assay	Technology	Time to result	Key targets	Sensitivity (Smear-neg)	Strengths	Limitations	NTEP scale-up
GeneXpert	Cartridge-based real-time PCR	<2 h	Mtb DNA, rpoB (RIF-R)	68–90%	Speed, automation, RIF-R detection	High cost, electricity-dependent	Widespread
Truenat	Chip-based micro-PCR	∼1 h	Mtb DNA, rpoB (RIF-R)	70–85%	Portable, battery-operated, affordable	Supply chain, training needs	Decentralized
Conventional PCR	Lab-based PCR	4–24 h	TB-specific genes	80–95%	High sensitivity, research utility	Contamination risk, lab-dependent	Reference labs

Smear microscopy is one of the oldest and most widely used diagnostic techniques for TB. It involves staining sputum samples to detect acid-fast bacilli (AFB), most commonly using the Ziehl-Neelsen (ZN) method. The lipid-rich cell wall of *Mycobacterium tuberculosis* resists decolourisation by acid-containing reagents, allowing visualisation under a microscope. Despite being rapid and inexpensive, smear microscopy suffers from poor sensitivity, ranging between 20%–80% depending on bacterial load. It is particularly inadequate in paucibacillary cases such as extrapulmonary TB, paediatric TB, or TB-HIV co-infection. Moreover, smear microscopy cannot distinguish live from dead bacilli, limiting its utility for monitoring treatment response or detecting drug resistance. Advances such as light-emitting diode (LED) microscopy and mercury vapour fluorescence microscopy have improved efficiency and sensitivity, with LED microscopy now recommended by WHO as a replacement for conventional fluorescence microscopy due to its durability and cost-effectiveness (31, 32).

Culture methods remain the gold standard for TB diagnosis and drug susceptibility testing. Solid media, such as Lowenstein-Jensen, and liquid culture systems, such as the Mycobacteria Growth Indicator Tube (MGIT), support bacterial growth and phenotypic analysis (33, 34). The proportion method, which compares colony growth on drug-containing vs. drug-free media, is widely used for DST and provides reliable results for first-line drugs such as isoniazid and rifampicin (35, 36). However, culture methods are slow, requiring 4–8 weeks for bacterial growth and an additional 4 weeks for DST, delaying treatment initiation (37). Automated liquid culture systems have improved turnaround times and reproducibility, but testing for second-line drugs remains less accurate (38). Other innovations, such as thin-layer agar, colourimetric redox indicator assays, and microscopic observation drug susceptibility assays, have been explored to provide faster and more precise DST results when WHO-endorsed assays are unavailable (39). Despite their limitations, culture methods are indispensable for confirming TB diagnosis, detecting drug resistance, and guiding treatment regimens (40).

### 8.2 Molecular diagnostics

Molecular diagnostics have become the first-line defence against TB infections because they can quickly, accurately, and specifically detect *Mycobacterium tuberculosis* and drug-resistance mutations (33). Unlike traditional methods such as smear microscopy or culture, which are slow and often lack sensitivity, molecular tools directly target bacterial DNA or RNA, thereby reducing the time to diagnosis (34). These technologies are especially important in countries with a lot of TB cases, like India, where early detection and drug-resistance profiling are necessary for effective treatment and TB elimination (6). The World Health Organisation (WHO) and the Centre for Disease Control and Prevention (CDC) support molecular diagnostics as first-line tools because they can detect TB and resistance to key drugs such as rifampicin (33, 41).

The GeneXpert MTB/RIF assay is a cartridge-based nucleic acid amplification test that simultaneously detects M. tuberculosis DNA and rifampicin resistance (42). Its mechanism is based on real-time PCR within a fully automated, closed system, which minimises contamination risks and simplifies laboratory workflow. The assay targets the rpoB gene, where mutations result in rifampicin resistance, and provides results in less than 2 h (41). GeneXpert is highly sensitive, even in smear-negative and HIV-related tuberculosis cases, and is widely used in India's National Tuberculosis Elimination Programme (NTEP) (6). Key advantages of GeneXpert include rapid turnaround, accuracy, and early detection of resistance; however, it is costly, requires stable electricity, and is limited in availability in remote regions (43). Despite these constraints, GeneXpert remains central to rapid tuberculosis diagnostics globally (33).

Truenat is a portable, chip-based molecular diagnostic tool developed in India to make tuberculosis testing more accessible in remote and low-resource areas (44). It uses micro-PCR technology to amplify TB-specific DNA and detect rifampicin resistance. The device is battery-powered and lightweight, delivering results in about an hour, making it practical for primary health centres and rural clinics (6). Truenat uses real-time amplification on a microchip and digital interpretation of results for accuracy and ease of use. Its low cost and suitability for resource-limited settings make it valuable for India's efforts to eliminate tuberculosis. However, there are still challenges with supply chains, staff training, and maintaining quality across different locations (45).

Polymerase chain reaction (PCR) tests, including both conventional and real-time PCR, detect tuberculosis by amplifying TB-specific DNA sequences (46). These methods are highly sensitive and can detect TB even when bacterial counts are low or when it occurs in parts of the body outside the lungs. PCR is especially useful in research and reference labs, where it supports detailed genetic studies and drug-resistance testing. The process uses repeated cycles to produce millions of copies of TB DNA, enabling accurate detection. However, PCR requires advanced lab equipment, trained staff, and strict contamination controls, which limit its use in resource-limited settings (33). Even with these challenges, PCR is still an important tool and works alongside GeneXpert and Truenat in broader TB detection efforts.

### 8.3 Emerging diagnostic technologies

New diagnostic technologies are improving TB detection through genomics, artificial intelligence, and biomarker-based methods. These approaches help address the limitations of traditional microscopy and culture, which are often slow and not very sensitive, as well as molecular tools like GeneXpert, which can be expensive and require specialised equipment (47). Since almost 90% of TB cases occur in resource-limited settings, researchers are developing faster, more affordable, and scalable diagnostic tools. Some recent advances include whole-genome sequencing (WGS) for detailed drug-resistance testing, AI-assisted radiology for automated chest x-ray interpretation, and new biomarker tests that improve accuracy for groups such as children and people with HIV (1). Together, these technologies aim to speed up diagnosis, improve accuracy, and make personalised treatment more accessible and affordable (29, 47).

Artificial intelligence (AI) is now an important tool for diagnosing TB, especially in interpreting chest x-rays. Machine learning, particularly convolutional neural networks (CNNs), can detect TB-related patterns in x-rays with high accuracy. Research shows that AI models can distinguish between drug-sensitive and drug-resistant TB and between active TB and latent infection. For instance, AI systems have achieved 84.3% sensitivity and 92.7% specificity in identifying TB cases, often outperforming traditional radiology (48). AI also enables remote diagnosis, as chest x-rays can be sent from mobile devices to central servers for automated analysis. This is especially helpful in rural or underserved areas. By reducing the need for radiologists, reducing human error, and enabling large-scale screening, AI-assisted radiology is becoming a key part of TB detection in resource-limited settings (49). Despite these advantages, the large-scale implementation of AI-assisted diagnostic systems in low-resource settings remains challenging. High initial setup costs, dependence on stable internet connectivity, variability in imaging quality, and limited availability of trained technical personnel may reduce the scalability and reliability of AI-based screening programmes in peripheral healthcare centers. In addition, concerns regarding algorithmic bias, data privacy, ethical use of patient information, and overreliance on automated interpretation require careful regulatory oversight. Therefore, AI technologies should complement rather than replace conventional clinical and radiological expertise within national TB control programmes.

Whole-genome sequencing (WGS) is a leading tool for diagnosing TB, as it provides detailed genetic information about *M. tuberculosis* quickly. Unlike culture-based methods that can take weeks, WGS can detect single-nucleotide changes, insertions, deletions, and repeats in just a few hours, helping predict drug resistance and identify strains (50). Common platforms include Illumina HiSeq/MiSeq and Ion Torrent, with Illumina offering sequencing at about $0.02 per million bases. WGS also helps track how TB spreads by mapping transmission networks, which is especially useful in areas with high TB rates (29). The US and UK now use WGS instead of traditional drug susceptibility testing (DST) for first-line drugs, and the WHO recommends it for finding drug-resistant mutations (1).

Recent advances in molecular diagnostics have further strengthened the capacity for rapid detection of DR-TB. The World Health Organization (WHO) has endorsed targeted next-generation sequencing (tNGS) as a valuable tool for identifying mutations associated with resistance to first-line and second-line anti-tubercular drugs. Compared with conventional phenotypic drug susceptibility testing, tNGS provides comprehensive resistance profiling within a shorter timeframe and can support individualized treatment strategies. Similarly, the Xpert MTB/XDR assay represents a significant advancement by enabling rapid detection of resistance to isoniazid, fluoroquinolones, ethionamide, and second-line injectable drugs directly from clinical specimens. These technologies have the potential to improve early detection and management of MDR-TB and XDR-TB in India (51). However, widespread implementation will require investments in laboratory infrastructure, trained personnel, quality assurance systems, and cost-effective integration within existing diagnostic networks.

Although WGS is highly accurate and comprehensive, its large-scale implementation in low-resource settings remains constrained by high operational costs, limited laboratory infrastructure, shortage of trained personnel, and challenges associated with bioinformatics data management. Maintaining advanced sequencing facilities and ensuring quality control may be particularly difficult in rural and resource-constrained regions. Furthermore, the cost-effectiveness and scalability of WGS-based surveillance programmes within national TB control systems require further evaluation before routine implementation. Nevertheless, ongoing reductions in sequencing costs and the development of portable sequencing technologies may improve future accessibility and integration into TB control strategies.

## 9 Treatment strategies for tuberculosis

The primary aim of tuberculosis treatment is to eliminate *Mycobacterium tuberculosis*, prevent relapse, and reduce transmission (52). The main approach uses several drugs that target different types of bacteria, including actively growing, semi-dormant, and those living in acidic or low-oxygen environments (53). Initially, the standard treatment starts with an intensive phase in which the bacterial load is reduced, followed by a continuation phase to eliminate all the remaining organisms. Directly Observed Therapy (DOT) remains important for ensuring patients take their medication, especially in countries with high TB rates (54). It is observed that there is a need for personalised treatment which should be adjusted for each patient, considering factors like HIV infection, age, pregnancy, and other health conditions (55). Globally, there is a focus on shorter courses, all-oral regimens, and new drugs to improve outcomes and reduce side effects (1).

A key part of TB management is distinguishing among drug-susceptible TB (DS-TB), latent TB infection (LTBI), and drug-resistant TB (DR-TB) (56). Treating LTBI helps stop it from turning into active TB, often using shorter courses of rifamycin-based drugs, such as the 3HP regimen (isoniazid and rifapentine once a week for three months) (57). DS-TB is usually treated with a six-month course of first-line drugs, while DR-TB needs longer and more complicated treatments with second-line medicines. Patient-centred care, including nutrition support, counselling, and digital tools to help patients adhere to their treatment, is becoming increasingly important (52). The WHO's 2025 guidelines stress the use of all-oral treatments and new drug combinations to replace harmful injectable drugs, especially for MDR-TB and XDR-TB. Latent tuberculosis infection (LTBI) means that *Mycobacterium tuberculosis* is present in the body but not causing symptoms, though it can become active later. Treating LTBI is key to preventing progression to active disease and reducing its spread (58). The WHO and CDC recommend several treatment options, with shorter rifamycin-based regimens now preferred because they are easier for patients to complete and have fewer side effects (57). Nowadays, the 3HP regimen (isoniazid and rifapentine once weekly for 3 months) and the 4R regimen (daily rifampicin for 4 months) is very common. Older regimens like 6H or 9H (daily isoniazid for 6 to 9 months) are still used when rifamycins cannot be administered. These treatments are especially designed for people at higher risk, such as those with HIV, young children exposed to TB, and patients on immunosuppressant drugs. Isoniazid works by blocking mycolic acid production, while rifamycins stop RNA polymerase, helping to clear latent bacteria (59, 60).

For drug-susceptible TB (DS-TB), the usual treatment uses four main drugs: isoniazid (INH), rifampicin (RIF), pyrazinamide (PZA), and ethambutol (EMB) (52). Treatment has two parts: an intensive phase with all four drugs for two months, then a continuation phase with just INH and RIF for four months (55). Different drugs have different modes of action, like INH blocks mycolic acid synthesis, RIF inhibits RNA polymerase, PZA disrupts energy metabolism in acidic environments, and EMB affects cell wall synthesis (61). This mix helps kill both active and semi-dormant bacteria. Sticking to the treatment plan is very important, so the WHO still recommends DOTS (Directly Observed Treatment, Short-course) to prevent resistance and relapse. When administered correctly, DS-TB treatment is effective in over 85%–90% of cases (62). DR-TB, especially MDR-TB and XDR-TB, needs longer and more complicated treatments. MDR-TB does not respond to at least INH and RIF, while XDR-TB is also resistant to fluoroquinolones and some second-line injectable drugs (63). The WHO now recommends shorter treatments lasting 9 to 11 months when possible, using drugs such as bedaquiline, linezolid, clofazimine, and fluoroquinolones. If these are not suitable, longer treatments of 18 to 24 months are used (6). The WHO also recommends using only oral drugs and stopping the use of toxic injectables like kanamycin, which can cause hearing loss. Even with these improvements, MDR-TB treatment works in about 60% of cases, compared to about 90% for DS-TB. This is mostly because of side effects, trouble sticking to the treatment, and limited access to drugs (1). Patient-centred care, including nutrition, counselling, and digital tools to help with adherence, is very important. It is also crucial to coordinate with HIV care and watch for side effects like heart rhythm problems, liver issues, and nerve damage (15).

The arrival of new drugs like bedaquiline, delamanid, and pretomanid has greatly changed how TB is treated, especially for MDR/XDR-TB (63). In M. tuberculosis, bedaquiline acts as a diarylquinoline which blocks ATP synthase enzyme, stopping the energy production and killing the bacteria. It works against both active and dormant forms, which helps clear stubborn infections. Bedaquiline is now part of the WHO-recommended treatments for MDR/XDR-TB and has been associated with improved success rates. However, it can cause QT prolongation, so patients need ECG monitoring. Delamanid is a nitroimidazole that blocks mycolic acid production, which is required for bacterial cell wall synthesis. It works against both active and inactive bacteria, especially in low-oxygen conditions. Delamanid is used in MDR-TB treatments, often together with bedaquiline and linezolid. Like bedaquiline, it can cause QT prolongation, and it is still hard to get in many places (64). Pretomanid is another nitroimidazole that acts in two ways: it blocks cell wall synthesis and generates reactive nitrogen species under low-oxygen conditions, which kill dormant bacteria. Pretomanid is a key component of the BPaL regimen (bedaquiline, pretomanid, and linezolid), which is effective for XDR-TB and MDR-TB cases that cannot tolerate other treatments (65). This new regimen is a big step forward, offering shorter, all-oral treatment with better results. However, linezolid can cause side effects like nerve damage and low blood counts, so patients need close monitoring (1). These new drugs have changed TB treatment by targeting how bacteria generate energy and survive, a property that older drugs could not. Adding them to WHO guidelines has started a new chapter in TB care, with shorter, safer, and more effective treatments for drug-resistant TB.

## 10 National tuberculosis elimination programme (NTEP)

**Figure 6:**
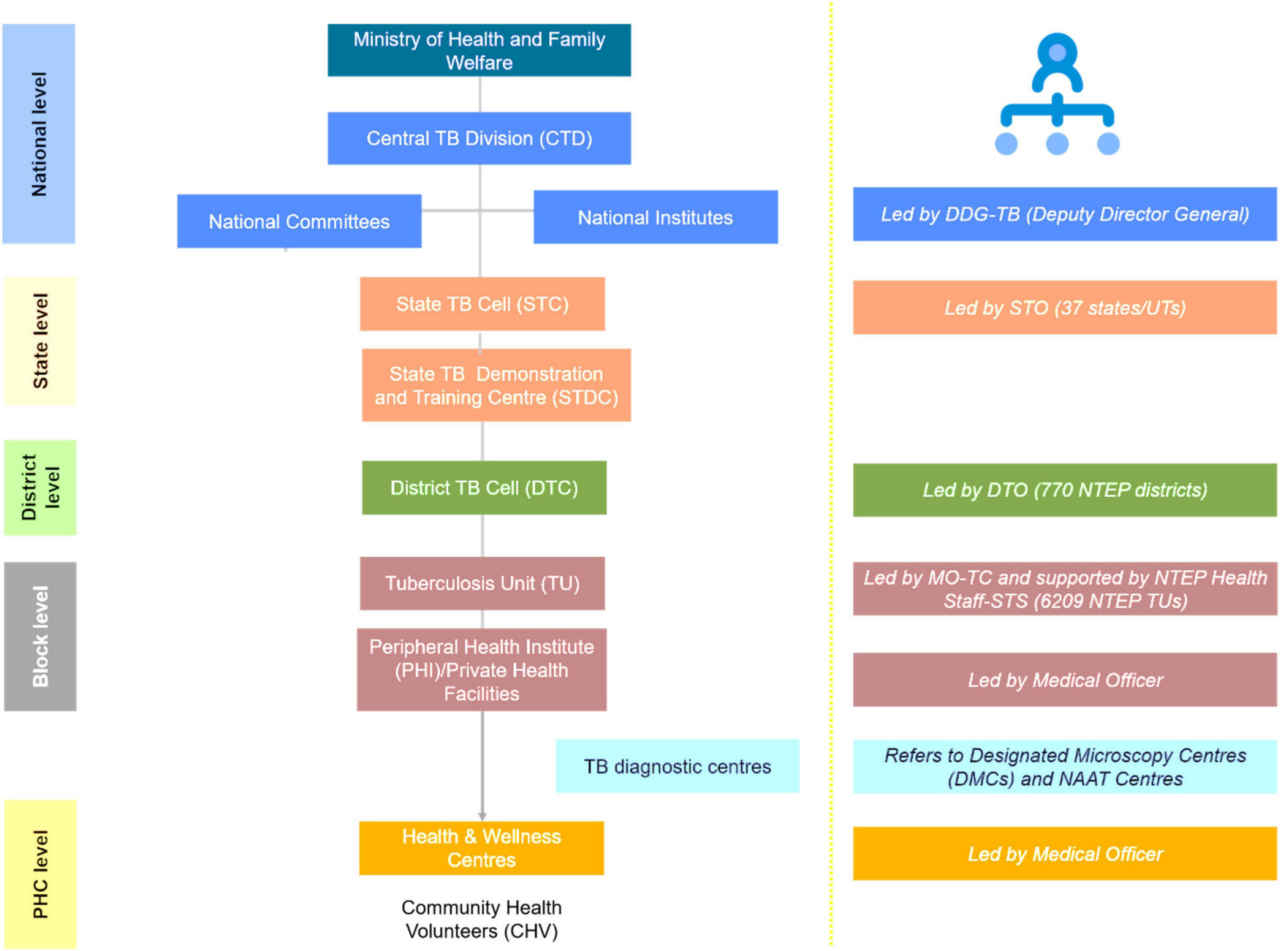
Organisational structure of India's national TB elimination programme (NTEP). Adapted from central TB division, ministry of health and family welfare (2023) (1, 64, 65).

The National Tuberculosis Elimination Programme (NTEP) represents India's primary public health strategy for achieving TB elimination by 2025, five years ahead of the global WHO End TB target of 2030. Originally implemented as the Revised National Tuberculosis Control Programme (RNTCP), the programme was restructured and renamed in 2017 to reflect a broader elimination-oriented approach (66). NTEP provides free diagnosis and treatment services, expands molecular diagnostic access through technologies such as GeneXpert and Truenat, integrates digital surveillance systems, and promotes community-based patient support initiatives (67). Given that India contributes nearly one-quarter of the global TB burden, the programme combines biomedical, digital, and social interventions to accelerate progress toward TB elimination (Figure 6) (1).

### 10.1 Programme structure and objectives

As illustrated in Figure 6, NTEP operates through a highly decentralised, multi-tier administrative structure extending from the national level to primary healthcare centres. The programme is coordinated centrally through the Central TB Division (CTD) under the Ministry of Health and Family Welfare and is implemented through State TB Cells, District TB Centres, Tuberculosis Units, and peripheral health institutions (68). This hierarchical structure enables nationwide implementation of surveillance, diagnosis, treatment, and reporting activities while supporting coordination between public healthcare systems, private providers, and local communities.

The organisational framework shown in Figure 6 also highlights the operational complexity of TB governance in India. Although decentralisation improves outreach and local implementation capacity, variations in infrastructure, workforce availability, funding distribution, and digital connectivity across states may contribute to inconsistent programme performance and reporting efficiency. In particular, coordination gaps between different administrative levels and between public and private healthcare sectors remain important barriers to timely diagnosis, case notification, and continuity of care. These structural challenges demonstrate that successful TB elimination depends not only on biomedical interventions but also on strengthening health systems governance, accountability, and implementation capacity across all administrative levels.

The objectives of NTEP are outlined in the National Strategic Plan (NSP) 2017–2025, which focuses on four major pillars: Prevent, Detect, Treat, and Build (69). The programme aims to reduce TB incidence by 80% and mortality by 90% relative to 2015 levels while ensuring that affected households do not experience catastrophic healthcare expenditure. Major interventions include universal drug-susceptibility testing, rapid molecular diagnosis, integration with HIV services, active case finding, nutritional support programmes, and digital monitoring systems (70). By combining medical treatment, surveillance, patient support, and community participation, NTEP attempts to establish a comprehensive and patient-centred framework for TB elimination in India (71).

### 10.2 Digital surveillance systems (Nikshay)

Nikshay is a cornerstone of NTEP and serves as India's national digital platform for tracking TB. Since its launch in 2012, Nikshay has been regularly updated to support real-time case reporting and monitoring in both public and private healthcare settings. The platform brings together patient records, test results, treatment tracking, and outcome monitoring to help ensure accountability and reduce the number of unreported cases (71). Nikshay also helps screen for both TB and COVID-19 and includes features for managing drug-resistant TB. Some recent additions are Nikshay SETU, a digital tool that helps healthcare providers learn and make decisions, and Ni-kshay Mitra, which links community donors with patients who need nutritional and emotional support (72). Nikshay makes it easier to use data for decision-making, improves programme accountability, and helps policymakers follow progress toward TB elimination goals. By moving surveillance online, India has made reporting more transparent, reduced delays, and improved coordination between public and private healthcare providers (73). Despite these advantages, the implementation of Nikshay continues to face several operational and governance-related challenges. Inconsistent data entry, underreporting from private healthcare providers, limited interoperability with state-level health databases, and variations in digital infrastructure across rural and urban regions affect the overall quality and reliability of surveillance data. In addition, concerns regarding patient privacy, digital literacy, and long-term sustainability of digital monitoring systems remain important barriers to effective implementation (74). Strengthening interoperability, ensuring standardized reporting practices, and improving digital health governance will be essential to maximize the effectiveness of Nikshay within India's TB elimination strategy.

### 10.3 Nutritional and patient support initiatives

Since malnutrition and TB are interlinked, NTEP includes nutrition checks, counselling, and financial help as part of patient care. Its main programme, Nikshay Poshan Yojana, provides TB patients with ₹500 per month to support nutrition. This support targets undernutrition, which is a major risk for TB to get worse, and helps patients stick to their treatment and recover (75). The Ni-kshay Mitra initiative also allows community donors, NGOs, and companies to provide additional nutrition, job training, and emotional support to TB patients. These patient-focused steps complement broader social protection programmes to provide care that extends beyond medical treatment (71). By bringing together medical treatment with nutrition and social support, NTEP tackles both the physical and social causes of TB. This helps patients get better results and face less stigma. India's approach shows that ending TB requires more than medical solutions alone, it also requires ongoing social and economic support for patients (1).

## 11 Challenges in TB elimination in India

India continues to pursue its ambitious goal of tuberculosis elimination by 2025; however, significant challenges related to disease burden, drug resistance, healthcare access, and social determinants remain (Table 3).

**Table 3:** National and global tuberculosis (TB) reduction targets, comparing India's 2015 baseline indicators with the sustainable development goals (SDG)/WHO end TB strategy targets for 2030 and India's accelerated elimination targets for 2025. The table highlights the ambitious reductions in TB incidence and mortality required to achieve TB elimination in India (1, 54).

Parameter	Base year-2015 (India)	2030 SDG/END TB target	India's commitment for 2025
Estimated annual incidence	217 cases/lakh	80% reduction compared to 2015	80% reduction compared to 2015 (44 cases/lakh)
Estimated annual Mortality	4.5 lakh	90% reduction compared to 2015	90% reduction compared to 2015 (Reduce to 45,000)

Unequal healthcare access is a big obstacle to ending TB. Although the NTEP has expanded, many rural and tribal areas still lack sufficient diagnostic and treatment centres. Advanced tests like GeneXpert and Truenat are mostly available in cities, so rural clinics rely on slower methods. Distance, poor infrastructure, and a lack of trained staff make quick diagnosis and treatment difficult. Stigma also prevents people, especially women and marginalised groups, from seeking care. TB services are not well linked to primary healthcare, which results in a poor completion rate. These problems delay case identification and increase the risk of TB transmission (1, 55). India's large private healthcare sector is another big challenge. About half of TB patients first go to private clinics or hospitals, but reporting from these providers is inconsistent. Many private doctors do not report cases to the national system (Nikshay), so some cases are missed and do not get NTEP support. Reasons include weak regulation, low awareness, and worries about patient privacy. Private doctors also sometimes use non-standard treatments or incomplete drug courses, which can make drug resistance worse. NTEP has made notification mandatory and set up digital platforms, but enforcement is still weak. Working more closely with private providers, encouraging them to report cases, and making sure they follow standard treatment are important steps (1, 73). These challenges also reflect broader governance and regulatory gaps within India's fragmented healthcare system. Weak enforcement of mandatory TB notification policies, limited incentives for private-sector engagement, and inadequate integration between public and private healthcare providers continue to undermine coordinated TB control efforts. Furthermore, variations in treatment practices and inconsistent adherence to national guidelines contribute to delayed diagnosis and increased risk of drug resistance (76). Addressing these systemic barriers will require stronger regulatory oversight, improved accountability mechanisms, and sustainable public–private partnership models grounded in health systems strengthening approaches.

Drug-resistant TB (DR-TB), especially resistance to isoniazid and rifampicin, makes treatment harder. DR-TB needs longer, more toxic, and more expensive treatments, with only about 60% success compared to about 90% for regular TB. XDR-TB, which is resistant to even more drugs, is even harder to treat. Causes include incomplete treatment, poor adherence, wrong prescriptions in the private sector, and limited access to drug-susceptibility testing. New drugs like bedaquiline, delamanid, and pretomanid help, but they are not widely available and need careful monitoring. Expanding drug-susceptibility testing, making new drugs more widely available, and supporting patients in sticking to treatment are key to preventing resistance (1, 55).

TB in India is strongly affected by social factors like poverty, malnutrition, overcrowding, and poor living conditions. Malnutrition both increases the risk of TB and can result from the disease, since it weakens the immune system. The Nikshay Poshan Yojana gives monthly nutrition support, but its reach and amount are limited. Crowded homes and poor ventilation help TB spread, especially in city slums. Stigma and discrimination make patients feel isolated and less likely to seek care or stick to treatment. Many patients stop treatment because they cannot afford to miss work or travel to clinics. These issues show that TB is closely tied to poverty and social inequality. Solving them requires action across sectors, including better housing, expanded nutrition programmes, social protection schemes, and community awareness campaigns (1).

Despite substantial progress under the National Tuberculosis Elimination Programme (NTEP), achieving tuberculosis elimination remains challenging due to persistent implementation gaps, including delayed diagnosis, underreporting from the private sector, treatment interruptions, healthcare workforce limitations, and socioeconomic determinants such as poverty, malnutrition, and overcrowding. The COVID-19 pandemic further exposed vulnerabilities in TB surveillance, diagnosis, and treatment services, resulting in missed cases and disruptions in continuity of care. These experiences highlight the importance of resilient health systems, integrated disease surveillance, and digital health infrastructure. Looking beyond 2025, sustained investments in rapid diagnostics, drug-resistance surveillance, public–private partnerships, social support programmes, and healthcare system strengthening will be essential to accelerate progress toward long-term TB elimination goals in India.

## 12 Future perspectives and strategies

Due to the limited effectiveness of conventional approaches in detecting and preventing TB, India is increasingly focusing on innovative strategies including advanced molecular diagnostics, novel vaccine development, and artificial intelligence-enabled digital health systems to accelerate progress toward TB elimination (1). Future TB elimination strategies should also incorporate implementation science and health systems strengthening frameworks to ensure effective translation of technological innovations into sustainable public health outcomes (74, 76). However, technological advancement alone will not be sufficient unless accompanied by improvements in healthcare accessibility, governance, surveillance quality, and antimicrobial stewardship.

Traditional culture-based diagnostic methods remain slow and less effective for early detection, particularly in resource-limited settings. Therefore, future strategies prioritise decentralisation of rapid molecular diagnostic platforms such as GeneXpert and battery-operated Truenat systems to improve accessibility in rural and underserved areas (6). Emerging technologies such as whole-genome sequencing (WGS) may further transform TB management by enabling rapid identification of drug-resistance mutations and personalised treatment approaches (29). In addition, biomarker-based diagnostic methods could improve diagnostic sensitivity in vulnerable populations including paediatric and HIV co-infected patients (47). Although current interventions largely focus on disease treatment and control, long-term TB elimination will also depend on the development of more effective vaccines capable of generating stronger immunity among adolescents and adults, who contribute substantially to community transmission (1).

Artificial intelligence and machine learning are also emerging as transformative tools in TB screening and surveillance. AI-assisted chest x-ray interpretation systems based on convolutional neural networks have demonstrated high diagnostic accuracy while enabling remote screening in resource-constrained settings (48). These technologies may reduce dependence on specialised radiologists and improve large-scale population screening capacity (49). Nevertheless, successful implementation of AI-driven healthcare systems will require standardised digital infrastructure, improved interoperability, ethical data governance, and adequate training of healthcare personnel to minimise disparities in technology access and utilisation.

**Figure 7:**
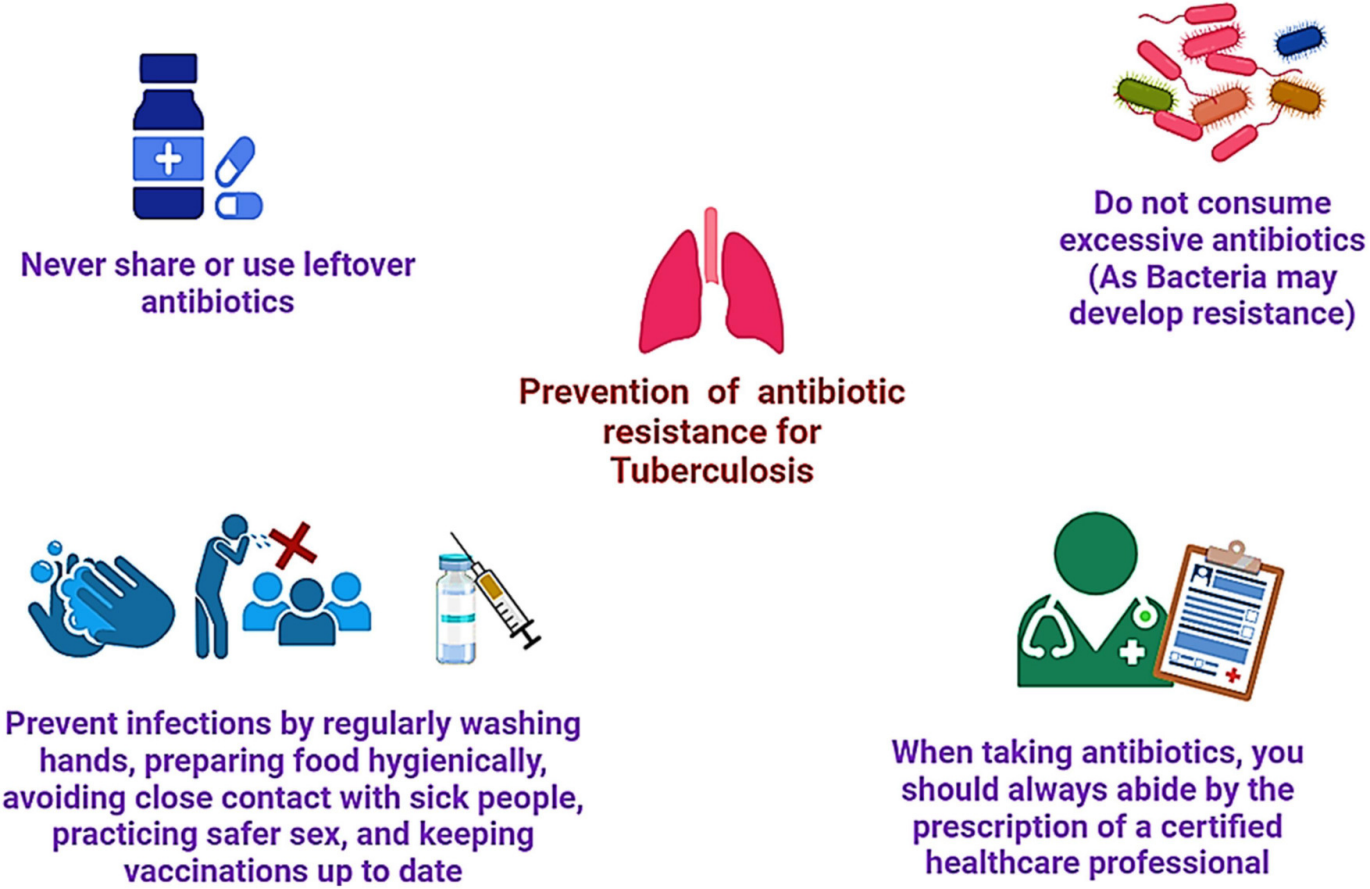
Administrative action plans to control antibiotic resistance/ multidrug resistance.

As illustrated in Figure 7, prevention of antibiotic resistance requires a multidimensional strategy extending beyond clinical treatment alone. Rational antibiotic use, adherence to prescribed therapies, infection prevention practices, vaccination, and public awareness are all essential components of antimicrobial stewardship. The figure further emphasises that prevention of drug resistance depends heavily on behavioural, healthcare, and public health interventions operating simultaneously at community and healthcare-system levels. In the context of TB, inadequate antibiotic regulation, incomplete treatment adherence, and misuse of antimicrobials may accelerate the emergence of multidrug-resistant and extensively drug-resistant strains, thereby threatening future treatment effectiveness.

To achieve the 2025 TB elimination target, India must adopt a coordinated and socially integrated public health approach that addresses major socioeconomic determinants including poverty, overcrowding, malnutrition, and unequal healthcare access (1). Programmes such as Nikshay Poshan Yojana and Ni-kshay Mitra should be further expanded to improve nutritional support, reduce financial burden, and enhance treatment adherence among vulnerable populations (71). Strengthening regulation and engagement of the private healthcare sector also remains critical, as a substantial proportion of TB patients initially seek care outside the public health system (11). Future policy efforts should therefore focus on strengthening public–private partnerships, improving mandatory case notification systems, standardising treatment practices, and integrating antimicrobial stewardship with broader TB control frameworks to minimise underreporting and prevent further emergence of drug resistance (Figure 7) (72, 73). To address these challenges, several initiatives have been implemented to strengthen private-sector engagement. The Private Provider Interface Agency (PPIA) model has facilitated standardized diagnosis, treatment, and reporting of TB cases through improved coordination between private practitioners and public health authorities. Incentive-based notification programs have further encouraged private providers to report TB cases, while integration with the Nikshay platform has improved case tracking, treatment monitoring, and data sharing. These initiatives have contributed significantly to strengthening TB notification and surveillance in India.

## 13 Conclusion

Tuberculosis remains one of the most significant public health challenges globally, with India continuing to account for approximately 26% of the worldwide TB burden. Despite considerable progress under the NTEP, the COVID-19 pandemic substantially disrupted TB diagnosis, treatment, surveillance, and patient access to healthcare services, resulting in temporary declines in case notification and the emergence of a potential hidden burden of undiagnosed TB cases. Although recovery in surveillance and treatment coverage has been observed in the post-pandemic period, persistent challenges continue to hinder India's progress toward TB elimination. The growing burden of multidrug-resistant tuberculosis (MDR-TB) and rifampicin-resistant tuberculosis (RR-TB) remains a major obstacle due to prolonged treatment duration, high treatment costs, drug toxicity, and limited accessibility of advanced therapies in resource-limited settings. At the same time, fragmented healthcare delivery systems, underreporting from the private healthcare sector, inconsistent treatment practices, and gaps in digital surveillance continue to contribute to ongoing transmission and antimicrobial resistance. Nevertheless, important advancements including expansion of molecular diagnostic platforms, implementation of digital surveillance systems such as Nikshay, adoption of all-oral treatment regimens, AI-assisted screening approaches, and patient-support initiatives have significantly strengthened India's TB response capacity.

Achieving India's ambitious 2025 TB elimination target will require a more integrated and strategically prioritised public health approach. In the short term, operational priorities should focus on strengthening early diagnosis, expanding rapid molecular and drug-susceptibility testing, improving treatment adherence, and enhancing the quality and interoperability of digital surveillance systems. Medium-term reforms should prioritise stronger public–private healthcare integration, expansion of antimicrobial stewardship programmes, improved healthcare infrastructure, workforce training, and equitable access to newer anti-tubercular therapies, particularly in rural and underserved regions. In the long term, sustainable TB elimination will depend on addressing broader structural and socioeconomic determinants including poverty, malnutrition, overcrowding, poor housing conditions, healthcare inequities, and social stigma, all of which continue to sustain disease transmission and worsen treatment outcomes. Ultimately, eliminating TB in India will require more than biomedical innovation alone. Sustainable progress will depend on coordinated multisectoral collaboration, strengthened health systems governance, evidence-based policymaking, social protection strategies, and sustained political and community commitment. Integrating technological innovation with patient-centred and equity-focused public health interventions will be essential to achieving long-term TB elimination and reducing the overall burden of disease in India.
